# Antarctic krill (*Euphausia superba*) oil modulatory effects on ethanol-induced acute injury of the gastric mucosa in rats

**DOI:** 10.3389/fnut.2022.1003627

**Published:** 2022-09-16

**Authors:** Luqiang Huang, Wenxin Wu, Linshan Huang, Jiaze Zhong, Lei Chen, Meiying Wang, Huibin Chen

**Affiliations:** ^1^College of Life Science, Southern Institute of Oceanography, Fujian Normal University, Fuzhou, China; ^2^The Public Service Platform for Industrialization Development Technology of Marine Biological Medicine and Product of State Oceanic Administration, Fujian Normal University, Fuzhou, China; ^3^Marine Active Substance and Product Technology Research and Development Center of Ocean Research Institute of Fuzhou, Fujian Normal University, Fuzhou, China; ^4^College of Food Science and Technology, Guangdong Ocean University, Zhanjiang, China; ^5^School of Engineering, University of Guelph, Guelph, ON, Canada

**Keywords:** Antarctic krill oil, supercritical carbon dioxide extraction, gastric mucosal injury, inflammation, polyunsaturated fatty acid

## Abstract

Antarctic krill oil (KO) prepared using supercritical carbon dioxide extraction and characterized using gas chromatography-mass spectrometry was used to investigate its preventive effect on ethanol-induced gastric tissue damage in a rat model *in vivo*. KO characterization showed that 74.96% of the unsaturated fatty acids consist of oleic acid, eicosapentaenoic acid (EPA), and docosahexaenoic acid (DHA). Rats pre-treated with KO (100, 200, and 500 mg/kg) showed mitigated oxidative stress through enhanced antioxidant enzyme superoxide dismutase (SOD) and reducing enzymes malondialdehyde (MDA) and myeloperoxidase (MPO) in gastric mucosal injury induced by ethanol. Additionally, the secretion of pro-inflammatory cytokines (TNF-α, IL-6, and IL-1β), the expression of the IκBα/NF-κB signaling pathway, and nitric oxide (NO) production was suppressed. The results also demonstrated a significant decrease in histological injury and hemorrhage scores in a dose-dependent manner in the KO range. Therefore, KO has potential as a food supplement to alleviate ethanol-induced acute gastric mucosal injury.

## Graphical Abstract

The work flow and the main findings of the KO modulatory effects. 
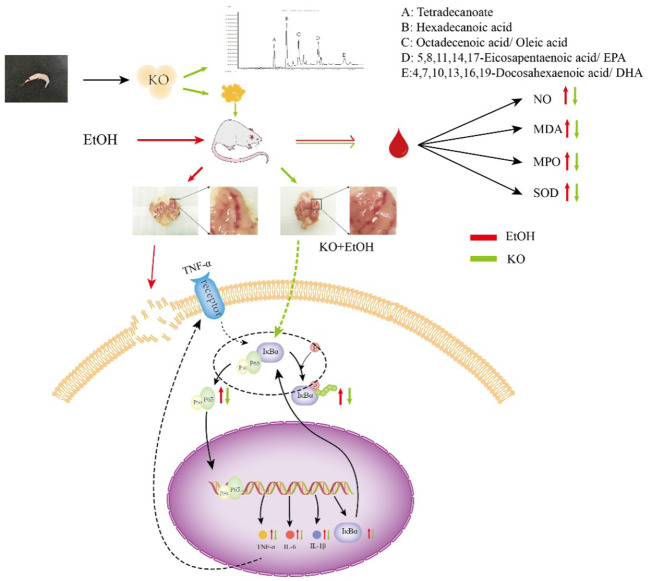

## Introduction

Gastrointestinal problems caused by alcoholism have become a global issue (1). Excessive alcohol intake is one of the most frequent causes of acute gastric mucosal injury, which may lead to gastric ulceration and bleeding and peptic perforation in severe cases (2). Alcohol causes histological damage to the mucosal layer, including hemorrhage and necrosis in local areas, necrosis and shedding of cells in the lamina propria of the mucosa, inflammatory cell infiltration, and the rupture and shedding of cells in the mucosal muscle layer (3). Advanced gastric ulceration typically requires active and long-lasting therapy, which causes an economic burden and is time-consuming for patients. Therefore, protecting the gastric mucosa against injury is vital for long term health (4).

As an exogenous toxic substance, the conversion of alcohol into intermediate products such as acetaldehyde, which can induce lipid peroxidation and oxidative stress, is the cause of acute gastric mucosal injury (5). For example, myeloperoxidase (MPO), which can generate the cell toxic substance hypochlorous acid (HOCl), is a chemical marker of neutrophil infiltration that reflects damaged tissue (6). Malondialdehyde (MDA), one of the end-products of lipid peroxidation, is used to evaluate the extent of lipid peroxidation in damaged tissues. Superoxide dismutase (SOD) has the function of scavenging oxygen free radicals and protecting cells from damage (7). The overexpression of nitric oxide synthase (NOS) produces excess NO, which is cytotoxic and causes gastric mucosal damage (8). Furthermore, ethanol-induced inflammation and excessive reactive oxygen species (ROS) production can impair DNA and lipid degradation, leading to irreversible cell damage (9). Because of the awareness of such problems, a new upsurge in the natural antioxidant and anti-inflammatory stuffs uptake in the diet to ameliorate gastrointestinal issues is being researched. Antarctic krill oil (KO) is an abundant source of unsaturated fatty acids, especially omega-3 fatty acids, such as eicosapentaenoic acid (EPA) and docosahexaenoic acid (DHA), and is as an important marine food additive (10, 11). Omega-3 fatty acids exert preventive and protective effects against oxidative stress and inflammation. Dietary intake of omega-3 fatty acids has been reported to increase the activity of antioxidant enzymes in an inflammatory disease mice model (12). Cheng et al. (13) showed that supplementation of omega-3 fatty acids in the form of EPA and DHA reduces oxidative stress and inflammation in the kidneys of rats with chronic renal failure and inhibits NF-κB activation. Donato-Trancoso et al. (14) found that oleic acid reduces oxidative damage and inflammation in rats, and EPA has been shown to reduce oxidative stress and inflammation in obese diabetic mice (15).

**Figure 1:**
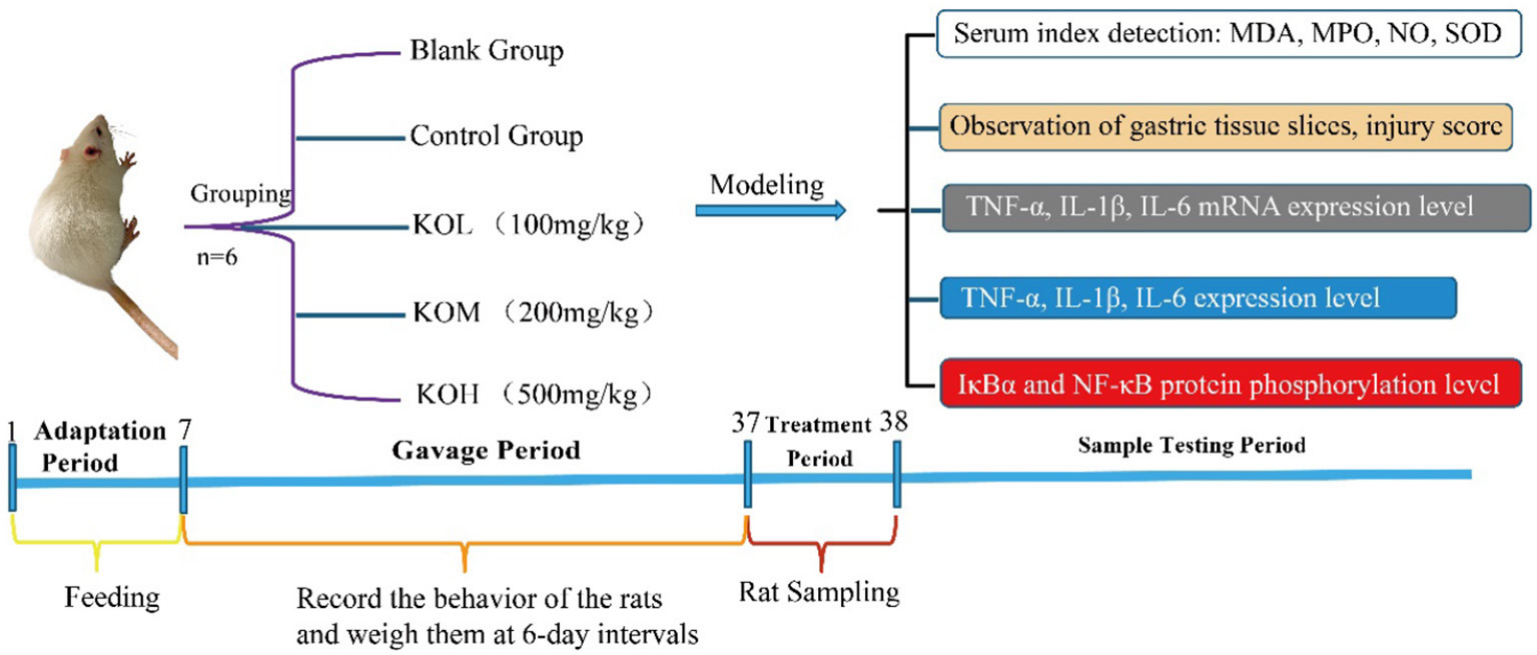
Experimental schematic flow chart of KO protection against ethanol-induced acute gastric mucosal injury.

The present study attempted to reveal that polyunsaturated fatty acids (PUFAs) in KO mice protect the gastric mucosa against ethanol-induced injury by anti-oxidation and inflammation. High-quality KO prepared through freeze-drying and supercritical carbon dioxide extraction (SFE) was orally administered to the ethanol-induced acute gastritis rat model in this study (Figure 1). Revealing the fundamental knowledge of gastric mucosal protection through KO will facilitate the exploitation and use of the natural resources of Antarctic krill as a functional food. The experimental scheme is shown in Figure 1.

## Materials and methods

### Materials

Antarctic krill was supplied by the Yangze Bait Industry Aquaculture Processing Enterprise (Luoyuan, Fujian, China), and BCA kits were purchased from Beyotime Biotechnology Company (Shanghai, China). Chemical reagents were provided by SIGMA (Sigma-Aldrich, St Louis, USA). P-IκBα, IκBα, NF-κB, P-NF-κB, and glyceraldehyde-3-phosphate dehydrogenase (GAPDH) antibodies were purchased from Cell Signaling Technology (Beverly, MA, USA).

### Preparation of Antarctic krill oil

Two methods, namely freeze-drying for 40 h and hot air drying to a constant weight, were used to prepare dried Antarctic krill. The KO extraction method was previous described (16) with some modifications: The dried krill was crushed into a powder using a grinder. KO was extracted from dried shrimp powder using a SFE device (Nantong Likai, China) with two steps of extraction and separation for 2 h. Carbon dioxide with 20 L/h flow rate passed through the dried shrimp powder at 35 MPa and 30°C in the extraction kettle, and then oil-gas separation at 4 MPa and 40°C in the separation kettle. KO samples were sealed and stored at 4°C until further use.

### Characterization of extracted krill oil

KO was collected from a separation kettle, and the component was characterized as described in a previous study (2). Briefly, methyl esterification of KO was performed using the following procedure: 0.1 g of KO mixed with 6 mL of methanol and hydrochloric acid (v:v, 1:19) was added to a test tube. The test tube was sealed and incubated in a water bath at 60°C for 2 h. An interval vortex was applied every 20 min to ensure full methylation. After purification with n-hexane, the oil was dissolved in 1.5 mL n-hexane (chromatographic grade) and processed through a 0.22 μm filter for further injection. Fatty acids were characterized using gas chromatography-mass spectrometry (GC-MS) (5977 B Agilent, State of California, USA) (Supplementary Figure S1). The experimental conditions were as follows: GC-MS detection, HP-88, 100 m × 0.250 mm × 0.20 μm; oven, 140°C for 5 min, gradually increased to 250°C at a rate of 4°C/min and maintained for 15 min.

### Determination of tocopherols, vitamin A, and astaxanthin

The tocopherols and vitamin A contents were determined using a Waters 2695 high-performance liquid chromatography (HPLC) system equipped with an ultraviolet detector (Waters 2487 2-channel UV/VIS detector, State of California, USA), according to the protocol described in a previous study (2). Briefly, 0.5 g of KO was mixed with ascorbic acid and KOH in ethanol, and saponified at 60°C for 1 h. After extraction and evaporation, the obtained residue was dissolved in n-hexane and filtered through a 0.22 μm porous membrane. The injection volume was 10 μL. The tocopherols (α, β, and γ) were separated in a column Si60 system (4.6 m × 250 mm × 5 μm) at 30°C. The mobile phase was n-hexane/tetrahydrofuran (v/v, 95/5) at a flow rate of 1.0 mL/min. Vitamin A was measured in a column XB-C18 system (4.6 m × 250 mm × 5 μm; Yuexu, Shanghai, China) at 35°C using a mobile phase of 98% methanol solution at a flow rate of 1.0 mL/min. The analysis wavelengths were 294 and 325 nm for tocopherols and vitamin A, respectively. The standard curve and sample determinations are provided in Supplementary Figures S2, S3. Astaxanthin was analyzed according to the method described by Xie et al. (2).

### Animal treatment procedures and ethics approval

Male Sprague-Dawley (SD) rats (age, 6 weeks; weight, 150 ± 10 g) were purchased from Wu Shi Laboratory Animals (Fujian, China). The experimental animals were acclimatized for 1 week to the environment and diet. All rats were kept at ambient temperature (22 ± 2°C) and photoperiod (12:12 h night/day cycle, lights on at 7:00 a.m.) conditions, with chow diet and distilled water *ad libitum*. SD rats were randomly divided into five groups (*n* = 6 per group): (1) control group (0.9% normal saline), (2) model group (0.9% normal saline), (3) low KO dose (KOL) group (100 mg/kg of KO), (4) medium KO dose (KOM) group (200 mg/kg of KO), and (5) high KO dose (KOH) group (500 mg/kg of KO). The control and model groups underwent gastric administration with 0.9% normal saline once daily, whereas the KO groups were administered 100 mg/kg, 200 mg/kg, and 500 mg/kg of KO, respectively. The rats were weighed once weekly, and weight changes and growth activities were recorded during the experiment. After 30 d of gavage, all rats were fasted for 24 h.

After fasting, ethanol was used to induce acute gastric mucosal injury, according to the methods described by Li et al. (17). Briefly, rats were orally gavaged with 80% ethanol (1.0 mL) to induce acute gastric mucosal injury (for the model, KOL, KOM, and KOH groups) or normal saline (for the control group) 1 h before sacrifice. The rats were anesthetized with 2% pentobarbital sodium and then sacrificed using cervical dislocation. The blood and gastric tissue were collected and stored at −80°C for further determination.

This experiment was strictly performed in accordance with the National Guidelines for the Care and Use of Laboratory Animals of China. The protocols for animal experiments were approved by the Fujian Normal University Animal Care and Use Committee (License No. 20180001).

### Serum physicochemical assays

Rat serum was collected for nitric oxide (NO), malondialdehyde (MDA), superoxide dismutase (SOD), and myeloperoxidase (MPO) tests. Determination was performed using MDA, MPO, NO, and SOD kits (Nanjing Jiancheng Institute of Bioengineering, Nanjing, China), following the manufacturer's protocols. The inflammatory cytokines IL-1β, IL-6, and TNF-α were tested using IL-1β, IL-6, and TNF-α kits (ExcellBio, Shanghai, China), according to the manufacturer's instructions.

### Pathological histology

The gastric mucosa of rats was dissected and cleaned with PBS. The gastric mucosa was fixed by soaking in 10% formaldehyde solution and subjected to routine dehydration, paraffin embedding, sectioning, and hematoxylin-eosin (HE) staining (Transgen Biotech, Beijing, China) to prepare slices, as described previously. The pathological surface of the gastric mucosa was then observed using an optical microscope. The degree of gastric mucosal injury was calculated as described by Laine and Weinstein (18), with slight modifications. Briefly, the degree of injury was graded from 1 to 4 depending on the length of the bleeding zone: 1 = 1–5 mm, 2 = 6–10 mm, 3 = 11–15 mm, and 4 = more than 15 mm. The degree of injury was graded from 1 to 2 depending on the width of the bleeding zone:1 = 1–2 mm, 2 = more than 2 mm. Each bleeding point represents one point. Subsequently, the gastric mucosal injury score, injury inhibition rate, and gastric injury area were calculated for each group. The following equations were used: injury score index = total score of group/number of animals in the group. Injury inhibition rate = (model group damage integral–dose group damage integral)/(model group damage integral) × 100%. Gastric injury area = π × d_1_ × d_2_ × ¼, where π is the circumference ratio, and d_1_ and d_2_ are the largest transverse and longitudinal diameters, respectively, measured through the center of the ulcer. Gastric injury incidence was calculated as follows: gastric injury area/total gastric area ×100%.

### Gene expression determination

RNA was extracted from the gastric tissues using an RNA extraction kit (Transgen Biotech, Beijing, China), and reverse transcription of RNA to cDNA was performed using a reverse transcription kit (SimpliAmp, Thermo Fisher Scientific, Massachusetts, USA). Real-time quantitative polymerase chain reaction (qPCR) analysis was performed using a qPCR kit (CFX96 BIO-RAD, California, USA). The expression of GAPDH was used as the internal standard for calibration. The experimental operations were based on the instructions provided with the kit and the experimental operations of Zhou et al. (19). The primers used in this study were obtained from Sangon Biotech (Shanghai, China) and are shown in Supplementary Table S1.

### Western blot detection of tissue inflammatory factor protein expression

Western blotting was used to analyze proteins from gastric tissues according to the methods described by Tu et al. (20). The primary antibodies used in this study were p-IκBα, IκBα, p-NF-κB, NF-κB, and GAPDH. The relative expression of the target protein was normalized to that of GAPDH, which was used as the internal reference.

### Statistics

The results were expressed as mean ± standard error of the mean (SEM) and analyzed using analysis of variance (ANOVA) and Dunn's test using SPSS 19.0 software. The graphing was performed using origin software (version 8.0). The gray value of the western blot was analyzed using ImageJ software (https://ij.imjoy.io/).

## Results

### Chemical characterization of krill oil

The SFE-extracted fatty acids of KO from freeze-dried and hot-air dried Antarctic krill were characterized (Table 1). The results showed that the fatty acid composition of the collected KO from hot-air-dried samples was 89.858 ± 3.014%, of which saturated fatty acids account for 22.48 and 67.38% unsaturated fatty acids content, with oleic acid, EPA, and DHA accounting for 15.92 ± 1.26%, 13.713 ± 1.587%, and 10.792 ± 1.639%, respectively. The KO from freeze-dried samples was 97.087 ± 2.345%, of which saturated fatty acids accounted for 25.04% and 74.96% unsaturated fatty acids, with a high content of oleic acid, EPA, and DHA accounting for 19.69 ± 1.37%, 14.76 ± 1.36%, and 11.17 ± 1.04%, respectively. The data showed that the content of unsaturated fatty acids in KO from freeze-dried samples was significantly higher than that in hot-air-dried samples. In addition, KO from freeze-dried samples had a high content of active components, including astaxanthin, α-tocopherol, and vitamin A, accounting for 589.00 ± 9.85 μg/g, 600 ± 14.3 μg/g, and 96.04 ± 4.7 μg/g, respectively. Therefore, KO from freeze-dried samples was used for further research.

**Table 1:** Characterization of SFE-extracted fatty acids using GC-MS.

**Fatty acids content (%)**	**Pre-treatment of Antarctic krill**	
	**Freeze drying**	**Hot air drying**
C12:0	0.225 ± 0.012	0.214 ± 0.015
C13:0	0.153 ± 0.02	0.122 ± 0.015
C14:0	7.129 ± 1.235	5.134 ± 0.789
C14:1	0.296 ± 0.031	0.265 ± 0.036
C15:0	0.628 ± 0.058	0.581 ± 0.026
C15:1	0.191 ± 0.014	–
C16:0	14.170 ± 2.123	14.484 ± 1.569
C16:1	6.188 ± 0.568	5.946 ± 0.478
C16:2	0.087 ± 0.012	0.702 ± 0.026
C16:3	0.320 ± 0.02	0.289 ± 0.032
C16:4	4.777 ± 0.569	3.427 ± 0.489
C17:0	0.780 ± 0.069	0.361 ± 0.058
C17:1	0.571 ± 0.064	1.193 ± 0.098
C18:0	1.567 ± 0.859	1.509 ± 0.678
C18:1	19.685 ± 2.365	15.916 ± 1.256
C18:2	3.463 ± 1.056	2.017 ± 0.769
C18:3	1.826 ± 0.689	1.984 ± 0.725
C18:4	4.274 ± 0.678	3.694 ± 0.852
C19:1	0.101 ± 0.012	0.420 ± 0.023
C20:0	0.292 ± 0.021	0.244 ± 0.011
C20:1	1.537 ± 0.063	1.896 ± 0.058
C20:2	0.154 ± 0.023	0.395 ± 0.032
C20:3	0.187 ± 0.042	0.404 ± 0.039
C20:4	1.390 ± 0.048	2.224 ± 0.074
C20:5	14.756 ± 1.369	13.713 ± 1.587
C22:1	0.869 ± 0.035	1.508 ± 0.246
C22:6	11.165 ± 1.036	10.792 ± 1.639
C24:0	0.087 ± 0.018	0.197 ± 0.056
C24:1	0.208 ± 0.026	0.218 ± 0.018
Total PUFA	42.402 ± 2.032	40.009 ± 1.698
Total FA	97.087 ± 1.345	89.858 ± 3.014

### Effect of krill oil on the pathological histology in ethanol-induced acute injury of the gastric mucosa in rats

**Figure 2:**
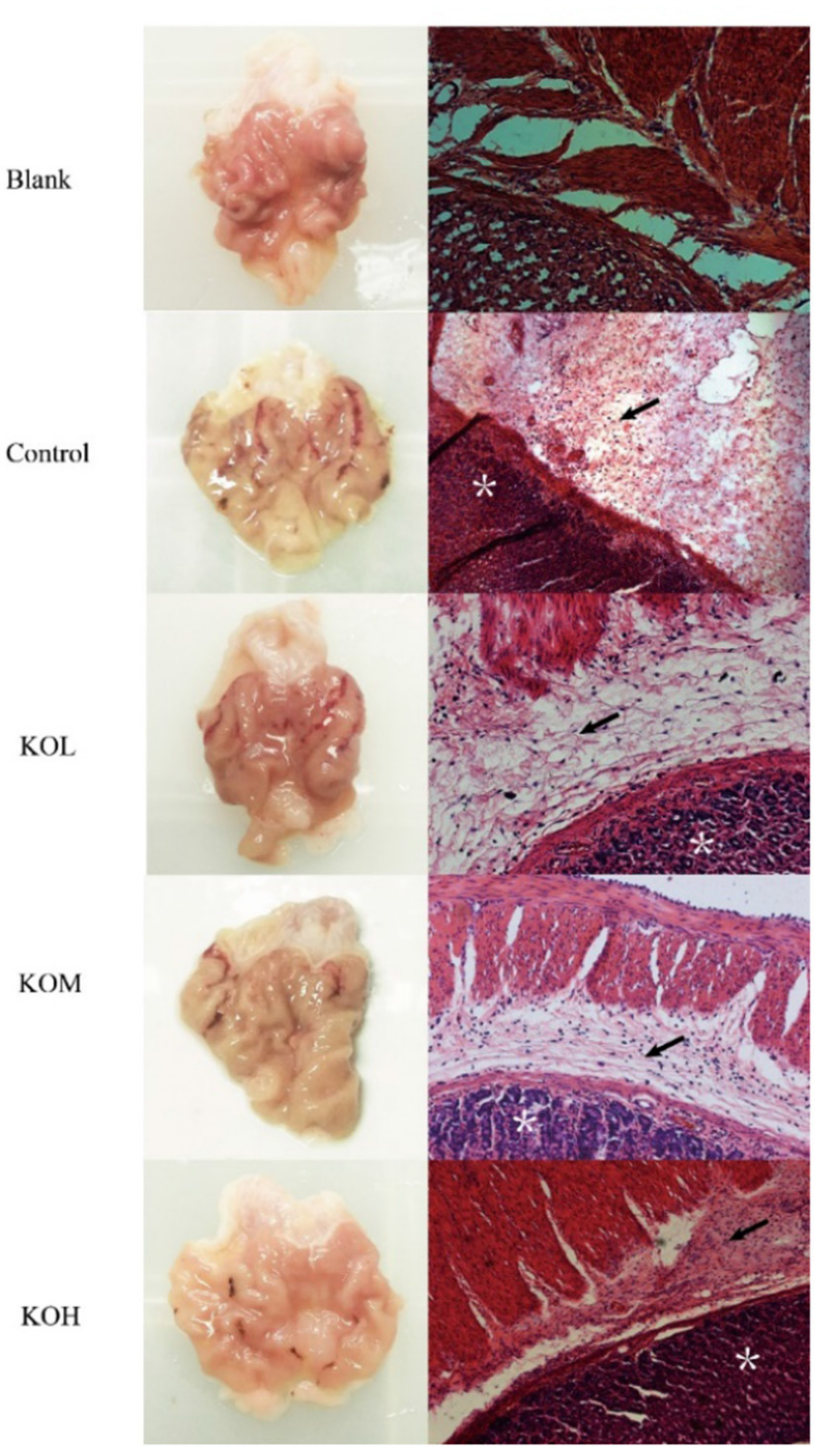
Effect of KO on the morphology and pathological histology of sections of the gastric mucosa. H&E staining of muscularis mucosa, epithelia, and lamina propria (×200) showed that the control section was severely injured, with extensive hemorrhage and necrosis in local areas, the necrosis and shedding of cells in the lamina propria of the mucosa, extensive inflammatory cell infiltration, and rupture and shedding of cells in the mucosal muscle layer. Following pre-treatment with different doses of KO, the damage of gastric mucosa was reduced in a dose-dependent manner. Arrows indicate the edema of submucosa and inflammatory infiltration. *Depicts damaged mucosal epithelium with disrupted glandular structure.

The pathological histology of the mucosal layer was examined in the rats (Figure 2). As seen in Figure 2, injuries in the model group occurred severely but were alleviated by KO pre-treatment at concentrations of 100, 200, and 500 mg/kg. Histological injury and hemorrhage scores were further assessed (Supplementary Table S2). The incidence of gastric injury in the ethanol group was 65.25%, and the injury integral index was 46. Compared with the model group, the gastric damage in the KO pre-treated groups was alleviated, in which KOL, KOM, and KOH were 50.56, 40.26, and 30.69%, respectively. The inhibitory rates of injury increased by 26.09, 43.48, and 54.34%, respectively. The damage integral indices for KOL, KOM, and KOH were 34, 26, and 21%, respectively. These changes alleviated acute ethanol injury in a dose-dependent manner.

### The effect of krill oil on the weight of rats

**Figure 3:**
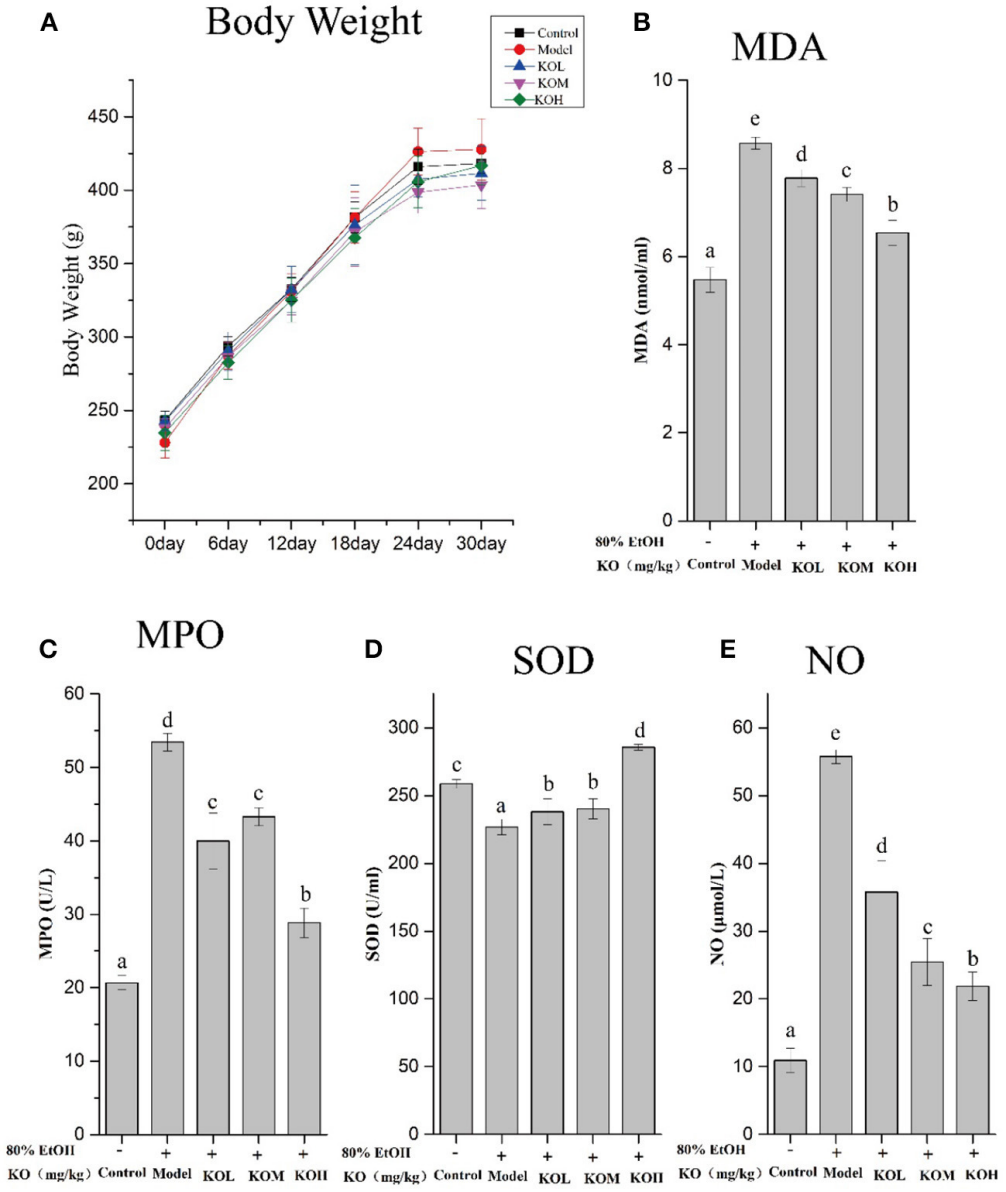
Levels of MDA, SOD, MPO, and NO in ethanol-induced gastric mucosal damage in SD rats after gavaging KO for 30 d **(A–E)**, and the weight changes of SD rats. Different letter values (*n* = 6; mean ± SEM) indicate statistically significant differences from the same column model group (*p* < *0.05*). KOL, krill oil of 100 mg/kg; KOM, krill oil of 200 mg/kg; KOH, krill oil of 500 mg/kg.

The weight changes of rats were recorded during the experiment during 30 d of gavage (Figure 3). As shown in Figure 3A, the effect of the KO dose on the weight change of SD rats after continuous gavage for 30 d is shown in Figure 3A. No significant difference in weight gain was observed among any groups, indicating that KO treatment may not cause obesity in rats (*p* > *0.05*). It indicated that KO with a dose of <500 mg/kg intragastric administration is appropriate for further gastric mucosal injury experiments because this dose range did not influence weight of rats.

### Effect of krill oil on lipid peroxidation and oxidative stress

To investigate ethanol-induced gastric mucosal damage in SD rats, lipid peroxidation and oxidative stress-related indicators were evaluated. As shown in Figures 3B–E, MDA and MPO, which are products of lipid peroxidation, significantly increased after the administration of absolute ethanol compared to the control (*p* < *0.05*). However, pre-treatment with KOL, KOM, and KOH significantly reduced the MDA and MPO levels in the serum compared with those in the model group (*p* < *0.05*). In the present study, SOD and NO were used to elevate the products of oxidative stress. After the administration of 80% ethanol, the serum of rats showed significantly reduced levels of SOD and increased levels of NO (*p* < *0.05*). However, pre-treatment with KO significantly increased SOD levels and reduced NO levels in a dose-dependent manner compared with the model group (*p* < *0.05*).

### Effect of krill oil on the expression of inflammatory factors TNF-α, IL-1β, and IL-6

**Figure 4:**
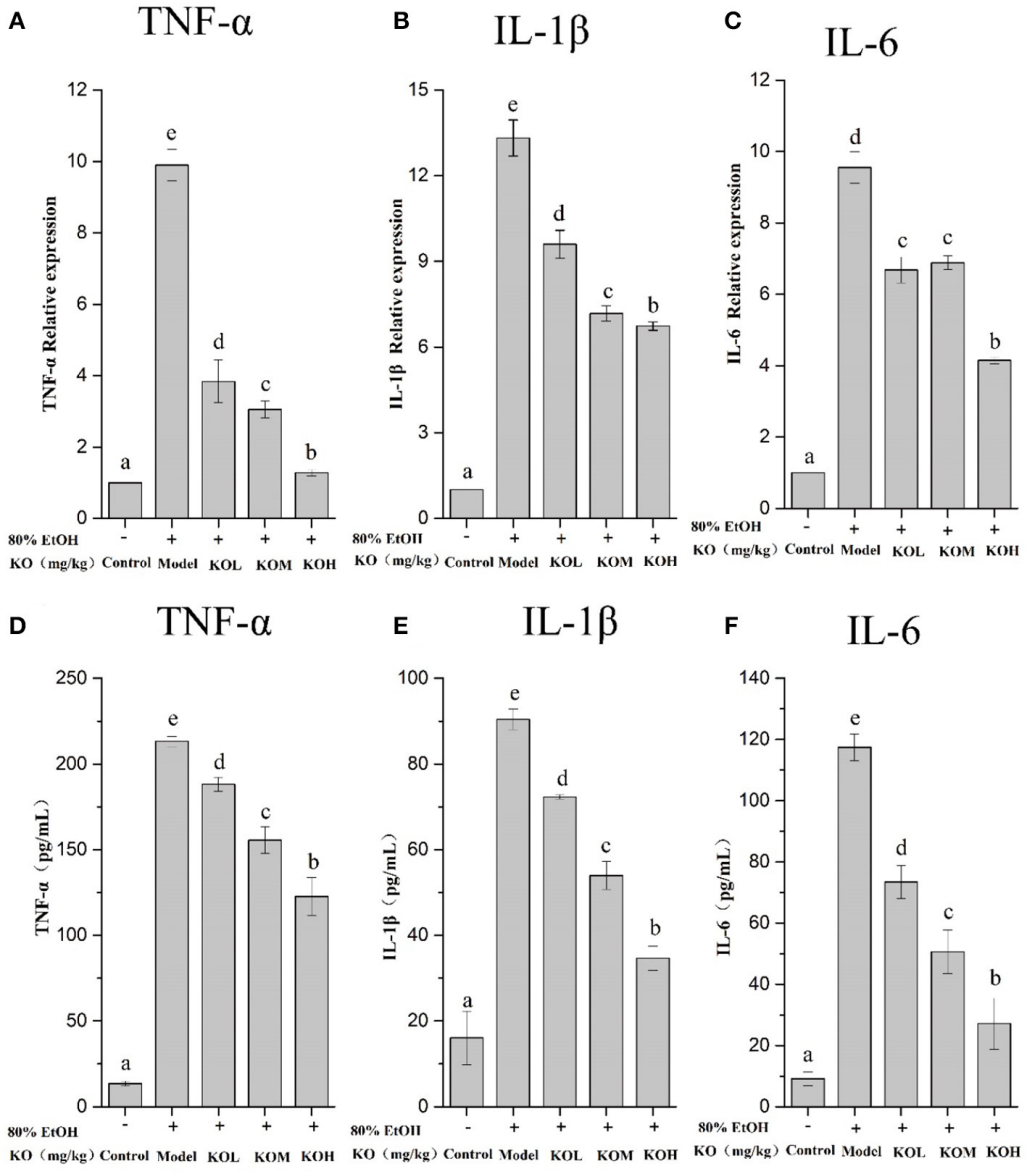
The expression of inflammatory factors was analyzed using qPCR **(A–C)** and the relative expression of inflammatory factors in tissues was detected using ELISA **(D–F)**. Different letter values (*n* = 6; mean ± SEM) indicate statistically significant differences from the same column model group (*p* < *0.05*). KOL, krill oil of 100 mg/kg; KOM, krill oil of 200 mg/kg; KOH, krill oil of 500 mg/kg.

The relative expression of tissue inflammatory factors was detected using qPCR, and the inflammatory factors (TNF-α, IL-1β, and IL-6) in rat serum were detected using enzyme-linked immunosorbent assay (ELISA); the results are shown in Figure 4. TNF-α in rat tissue of the model group significantly increased by 9.9 times compared to that in the control at the mRNA level (*p* < *0.05*). In the KOL, KOM, and KOH groups, the expression levels of TNF-α were 3.84, 3.06, and 1.27 times higher, respectively, which significantly decreases the expression of TNF-α in a dose-dependent manner (Figure 4A) (*p* < *0.05*). Similar to TNF-α, IL-1β and IL-6 showed decreasing trends with increasing doses of KO (Figures 4B,C). The above data show that KO significantly reduced the mRNA transcription levels of the pro-inflammatory factors TNF-α, IL-1β, and IL-6 in a dose-dependent manner (*p* < *0.05*). Similarly, ELISA detection for the secretion of TNF-α, IL-1β, and IL-6 in the serum of the model group significantly increased (Figures 4D–F) (*p* < *0.05*). The secretion of inflammatory factors such as TNF-α, IL-1β, and IL-6 significantly decreased with pre-treatment with KOs. The modulatory effect of inflammatory factors showed dose-dependency (*p* < *0.05*).

### Inhibitory effect of krill oil on IκB/NF-κB signaling pathway proteins in ethanol-induced acute injury of the gastric mucosa in rats

**Figure 5:**
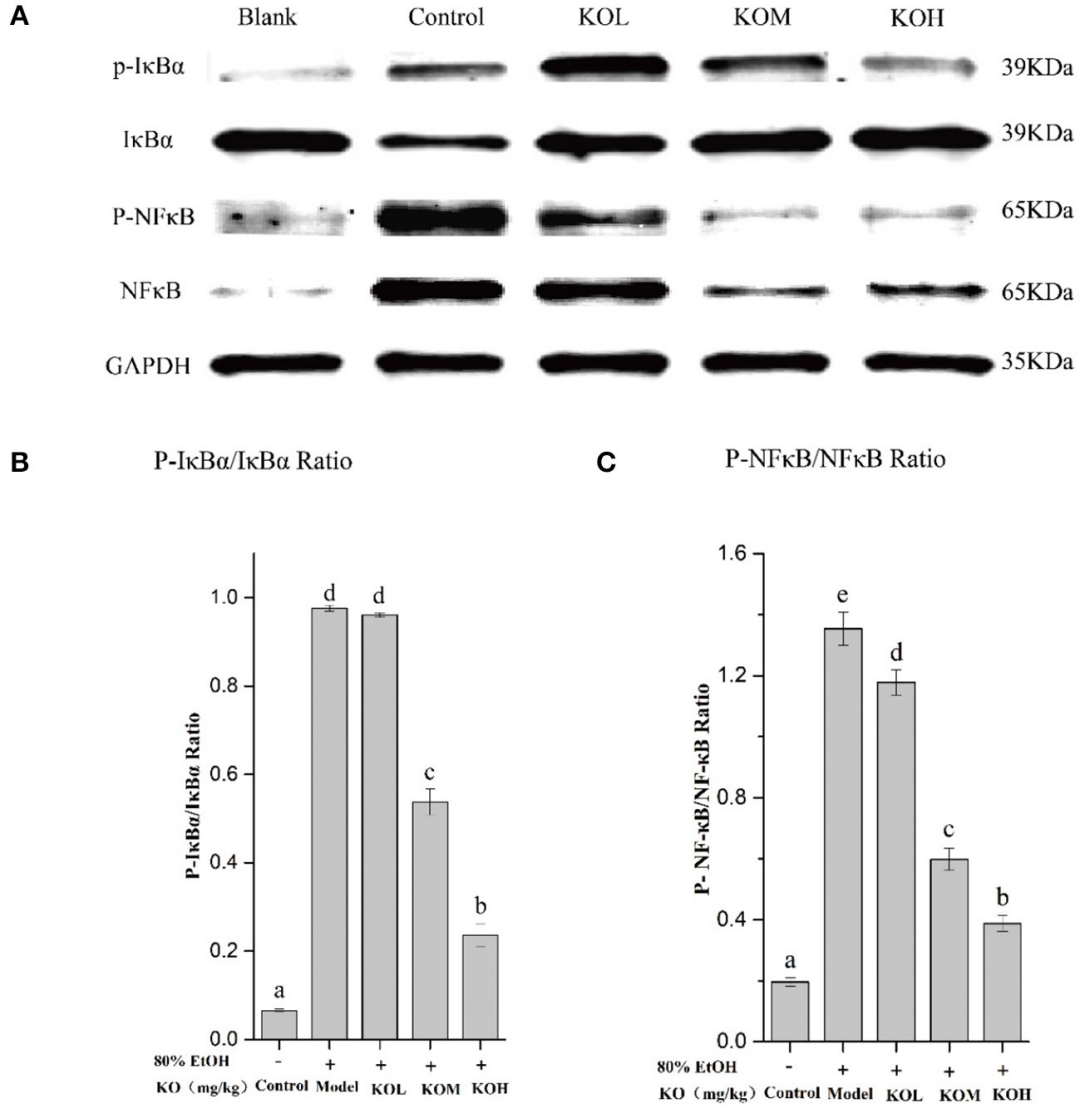
KO inhibits inflammation in the expression levels of IκBα and NF-κB in gastric tissues. (A) Protein levels of p-IκBα, IκBα, P-NF-κB, and NF-κB in colon were investigated using western blot analysis; (B) relative expression of P-IκBα/IκBα; (C) relative expression of P-NF-κB/NF-κB indicate that KO can significantly reduce inflammation by inhibiting the phosphorylation of IκBα and NF-κB. Different letter values (n = 6; mean ± SEM) indicate statistically significant differences from the same column model group (*p* < *0.05*). KOL, krill oil of 100 mg/kg; KOM, krill oil of 200 mg/kg; KOH, krill oil of 500 mg/kg.

As shown in Figure 5, the phosphorylation level of IκBα in the gastric tissue of the ethanol-induced model group significantly increased compared to that in the control (*p* < *0.05*). However, pre-treatment with KOM and KOH significantly reduced the phosphorylation level of IκBα in the gastric mucosa compared to that in the model group (Figures 5A,B) (*p* < *0.05*). When IκBα is degraded, the NF-κB protein is activated. Compared to the control, the phosphorylation level of NF-κB in the model group was significantly upregulated. However, pre-treatment with KOL, KOM, and KOH reduced the phosphorylation level of NF-κB in a dose-dependent manner (Figures 5A,C).

## Discussion

In this study, KO was extracted from frozen dried krill using the SFE method and purified KO was obtained. KO had a high content of unsaturated fatty acids with 19.69% oleic acid, 14.76% EPA, and 11.17% DHA. The contents of EPA and DHA were similar to a previous report in which the fatty acids of KO were extracted using organic solvents (2). However, SFE approximately produces 7% more oleic acid than organic chemical solvent extraction, which demonstrates the superiority of the method used in this study. This may be attributed to the high affinity of SFE for fatty acids, and oleic acid has the greatest solubility in supercritical carbon dioxide compared to other fatty acids such as linoleic acid, palmitic acid, and stearic acid (experimental measurement and correlation of phase equilibria of palmitic, stearic, oleic, linoleic, and linolenic acids in supercritical carbon dioxide). EPA and DHA can reduce oxidative stress and inflammation in the kidney of rats, and oleic acid plays a significant role in preventing gastric ulcerogenesis (13). Additionally, KO has been reported to effectively increase adiponectin levels and reduce endocannabinoid plasma levels, and palmitoleic acid in KO can help improve glucose homeostasis (21).

Excessive alcohol consumption can cause gastrointestinal problems such as acute gastric mucosal injury and gastritis (4). With ethanol stimulation, both oxidative stress and inflammation signaling pathways were activated in the gastric mucosa. This process generates ROS and proinflammatory cytokines, which can induce gastric injury (9).

Our histological evaluation showed that severe gastric damage occurred in ethanol-treated rats and was mitigated in the KO treatment in a dose-dependent manner. Inordinate production of oxygen free radicals leads to lipid peroxidation, an important marker of cell membrane oxidative damage induced by hydroxyl free radicals. Lipid peroxidation amplifies the role of ROS, increasing oxidative damage and accelerating the development of inflammation (22). Oxidation is a critical step in the pathogenesis of gastric ulcers. Antioxidants trap free oxygen radicals, thereby alleviating oxidative stress and lipid peroxidation. Therefore, these substances are viewed as potential antigastric ulcerogenic agents (23). As demonstrated, KO rats with a high content of unsaturated fatty acids showed significant protective effects against acute ethanol-induced gastric injury in our experiment. In previous reports, oleic acid exhibited effective anti-oxidative and anti-inflammatory (14) effects, because oleic acid can downregulate COX-2 and iNOS through the NF-κB signaling pathway to ameliorate inflammation (24). Additionally, both EPA and DHA can distinctly influence the monocyte inflammatory response, and DHA has a broader effect in attenuating pro-inflammatory cytokines than EPA (25). This may be attributed to DHA having versatile anti-inflammatory pathways through which both necrosis and ERK1/2 phosphorylation in THP-1 monocytes can be reduced by regulating the RIPK1/RIPK3 signaling pathway (26). In agreement with fish oil, high levels of DHA and EPA have been reported to significantly regulate inflammation by reducing the NF-κB and IL-1β expression levels (27).

Furthermore, the hydrophobic nutraceutical components, including vitamin E, vitamin A, and astaxanthin, were identified in KO extracts in significant quantities. Both vitamin A and its derivative, retinoic acid, have been reported to alleviate inflammation *via* different signaling pathways (28). Vitamin E is an antioxidant that contains four tocopherols and four tocotrienols. In addition, the combination of α- and γ-type tocopherols can better regulate inflammation than the antioxidant alone (29). Interestingly, it was found that only α-tocopherol was highly prevalent in the KO rats. High-purity astaxanthin has been reported to exert strong anti-inflammatory effects in patients with functional dyspepsia, because it specifically suppresses NO generation, which is connected to TNF-α secretion (30). The commercial products and shrimp waste extracts of astaxanthin have both NO and free radical O^−2^ suppression, suggesting that the extracts without the purification multicomponent may result from multiple functions (30). In addition, the functional advantages of combining astaxanthin and vitamins have been illustrated in previous studies. Hydrophobic antioxidants loaded into nanostructured lipid carriers have been reported to improve the chemical stability of astaxanthin embedded (31). In line with previous reports (20), lipid peroxidation, a critical marker involved in the generation of oxidative damage in ethanol-induced gastric mucosa, was observed in this study. Ethanol-induced oxidative stress in the gastric mucosa increased MDA and MPO levels and decreased SOD levels. However, pre-treatment with KO improved the antioxidant enzyme activity of SOD and reduced lipid peroxidation against acute gastric injury induced by ethanol. Therefore, KO alleviates ethanol-induced gastric injury by reducing oxidative stress.

In our experiment, oral ethanol administration stimulated the overproduction of the inflammatory mediator NO, which led to gastric mucosal injury. This evidence of NO from the overexpression of iNOS induced by ethanol is similar to that in previous reports (9). The overexpression of nitric oxide synthase (NOS) produces excess NO, which is cytotoxic and causes gastric mucosal damage (8). It was revealed that the levels of cytokines (TNF-α, IL-6, and IL-1β) significantly increased in ethanol-induced gastric injury. However, pre-treatment with KO extract effectively suppressed these inflammatory mediators in ethanol-induced gastric mucosal injury. TNF-α exhibits positive feedback in regulating the inflammatory response by binding to the TNF-α receptor and activating the NF-κB pathway to produce and release more TNF-α, thereby amplifying the inflammatory signal (32). The IκB/NF-κB signaling pathway is a classic inflammatory pathway that plays a vital role in various biological processes. It can induce a variety of transcription factors that are activated by proinflammatory cytokines. NF-κB is a key regulator of inflammatory response (33). Li et al. (1) reported that an increase in NF-κB expression increased the expression of inflammatory cytokines in gastric ulcers. Activation of the NF-κB signaling pathway is one of the key mechanisms by which cells respond to acute infection and inflammation (34). KO protecting against gastric mucosal injury in our study can be attributed to the high content of antioxidants, which was reported to reduce ROS-stimulated NF-κB activation (35) and inhibit ethanol-induced ulcer enlargement. The NF-κB family is considered to be the most widely studied target of inflammatory response due to the triggering of chain reaction (36), and is tightly linked to inflammation, immune response, cell cycle and cell survival (34). In addition, the activation of the NF-κB signaling pathway promotes the expression of other pro-inflammatory genes, such as IL-1β and IL-6. Zhou et al. (37) demonstrated that mitochondria are the source of ROS release, which stimulates the inflammasome and triggers the secretion of IL-1β, which is a lymphocyte-stimulating factor, and its content level reflects the severity of tissue inflammation in the body. IL-6 is another important cytokine as high levels of IL-6 activate a variety of cells in inflammatory lesions, such as neutrophils.

**Figure 6:**
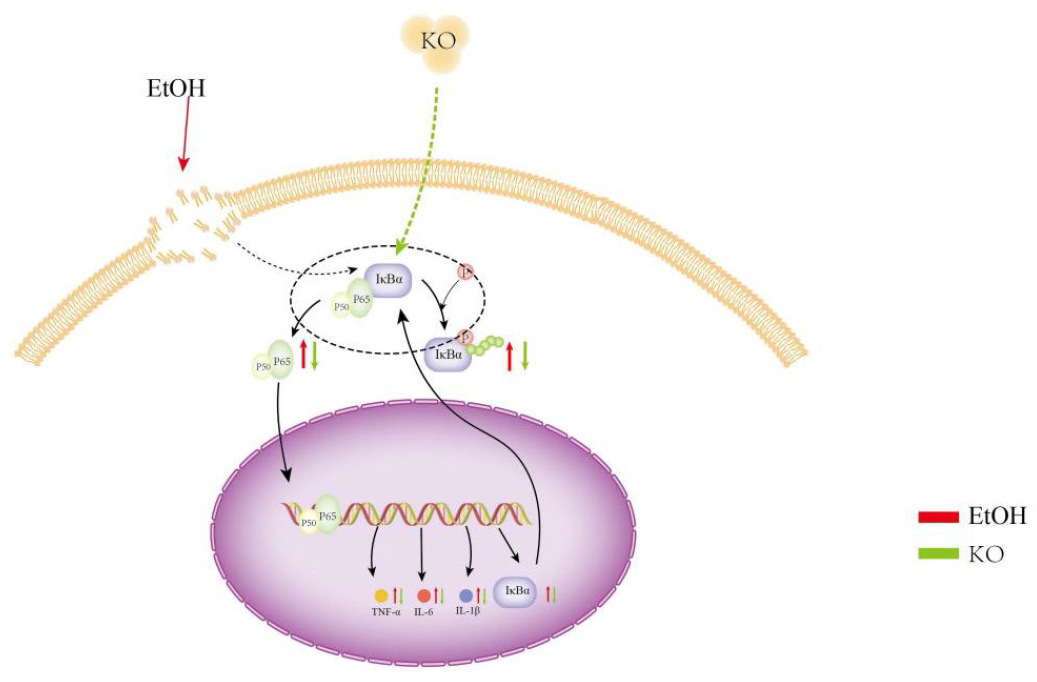
Protective effect of KO on gastric mucosa injury suffered from ethanol and subsequent inflammatory mediators. Under the induction of ethanol, gastric tissue generates a large number of ROS, which causes oxidative stress. Oxidative stress causes lipid peroxidation and an inflammatory reaction. Lipid peroxidation and inflammation further aggravates oxidative stress. Therefore, in this study, oxidative stress and lipid peroxidation increased MDA, NO, and MPO content in the control, whereas the content of SOD decreased. The activation of the NF-κB signaling pathway and the high production of inflammatory factors TNF-α, IL-1β, and IL-6 caused severe inflammation. Oxidative stress, lipid peroxidation, and inflammation interact to promote gastric mucosal injury and gastritis and lead to gastric ulcers and perforation.

In line with cytokines, hemorrhagic gastric mucosal damage and inflammatory cell neutrophil infiltration were verified through histological observations of ethanol-induced gastric tissue. Thus, KO had a protective effect against gastric mucosal injury caused by ethanol and subsequent inflammatory mediators. Zhang et al. (38) have shown that KO improves motor abnormalities and cognitive deficits in mice with multiple sclerosis, reduces vanillone-induced oxidative stress, and reduces the expression level of the NF-κB protein. Inhibiting the activation of NF-κB signal transduction is essential for further understanding the regulation of this signaling pathway and for developing new treatment strategies to improve gastric inflammation. In our experiment, KO robustly exhibited a regulatory effect in the initial and pre-relief stages of inflammation and protected the gastric mucosa against ethanol-induced acute injury (Figure 6). The full benefits of long-term intake in KO may be due to the synergy of multiple metabolic and signaling pathways.

## Conclusion

In summary, KO prepared using the SFE method from freeze-dried Antarctic krill contained a high level of multicomponent antioxidants such as unsaturated fatty acids (oleic acid, EPA, and DHA) and hydrophobic nutraceutical components, including vitamin E (α-tocopherol), vitamin A, and astaxanthin. These bioactive substances are responsible for the main protective effects of alleviating ethanol-induced gastric injury by reducing oxidative stress. In line with the observation of hemorrhagic gastric mucosal damage and inflammatory cell neutrophil infiltration, the secretion of inflammatory cytokines (TNF-α, IL-6, and IL-1β) in the gastric mucosa of rats was effectively suppressed by pre-treatment with KO extracts. Our experimental results demonstrated that KO pre-treatment mitigates ethanol-induced acute gastric mucosal injury in a rat model, suggesting the potential use of KO as a functional food for acute gastric mucosal injury and disorders.

## Data availability statement

The original contributions presented in the study are included in the article/Supplementary material, further inquiries can be directed to the corresponding author/s.

## Ethics statement

The animal study was reviewed and approved by Fujian Normal University Animal Care and Use Committee (License No. 20180001). Written informed consent was obtained from the owners for the participation of their animals in this study.

## Author contributions

HC: data checking, writing—review, revise, methodology, analysis, and discussing. MW: methodology, experimental design, analyzsis, and discussing. LuH: writing—original draft, methodology, analyzed, and discussed. WW performed the experiment, illustrating, checked the data, writing—review, and revise. LiH performed the experiment and statistical analysis. JZ performed the experiment, illustrating, and statistical analysis. LC: illustrating, writing—review, and revise. All authors read and approved the final version of the manuscript.

## Funding

This work was supported by the projects of the Distinguished Professor of Minjiang Scholar program (2019) and the scientific research innovation program Xiyuanjiang River Scholarship of College of Life Sciences, Fujian Normal University.

## Conflict of interest

The authors declare that the research was conducted in the absence of any commercial or financial relationships that could be construed as a potential conflict of interest.

## Publisher's note

All claims expressed in this article are solely those of the authors and do not necessarily represent those of their affiliated organizations, or those of the publisher, the editors and the reviewers. Any product that may be evaluated in this article, or claim that may be made by its manufacturer, is not guaranteed or endorsed by the publisher.
