# Supplementary material for: Antarctic krill (Euphausia superba) oil modulatory effects on ethanol-induced acute injury of the gastric mucosa in rats
Source: Front Nutr. 2022 Sep 16;9:1003627. doi: 10.3389/fnut.2022.1003627 (PMC9525105; doi:10.3389/fnut.2022.1003627)
Supplement: Supplementary file 1 [file Data_Sheet_1.docx]

**Supplementary Information**

**Antarctic Krill (*Euphausia superba*) Oil Modulatory Effects on Ethanol-Induced Acute Injury of the Gastric Mucosa in Rats**

Luqiang Huang ^a,b,c^, Wenxin Wu ^a^, Linshan Huang ^a^, Jiaze Zhong ^a^, Lei Chen ^d^, Huibin Chen ^a,b*^ and Meiying Wang ^e**^

^a^ Southern Institute of Oceanography, College of Life Science, Fujian Normal University, Fuzhou 350117, China

^b^ The Public Service Platform for Industrialization Development Technology of Marine Biological Medicine and Product of State Oceanic Administration, Fujian Normal University, Fuzhou 350117, China

^c^ Marine Active Substance and Product Technology R&D Center of Ocean Research Institute of Fuzhou, Fujian Normal University, Fuzhou 350117, China

^d^ College of Food Science and Technology, Guangdong Ocean University, Zhanjiang 524088, China

^e^ School of Engineering, University of Guelph, Ontario N1G 2W1, Canada

E-mail address:

Luqiang Huang: [biohlq@fjnu.edu.cn](mailto:biohlq@fjnu.edu.cn); Wenxin Wu: [Wuwx1825016@163.com](mailto:Wuwx1825016@163.com); Linshan Huang: 1484253160@qq.com; Jiaze Zhong: zhongjiaze@amoytop.com; Lei Chen: chenlei841114@hotmail.com; Huibin Chen: chuibin@fjnu.edu.cn; Meiying Wang: [mwang15@uoguelph.ca](mailto:mwang15@uoguelph.ca)

* Corresponding author: Huibin Chen

Fax: +86 591-22868200

E-mail address: chuibin@fjnu.edu.cn; vipin_chen@163.com

* * Co-corresponding author: Meiying Wang

E-mail address: mwang15@uoguelph.ca


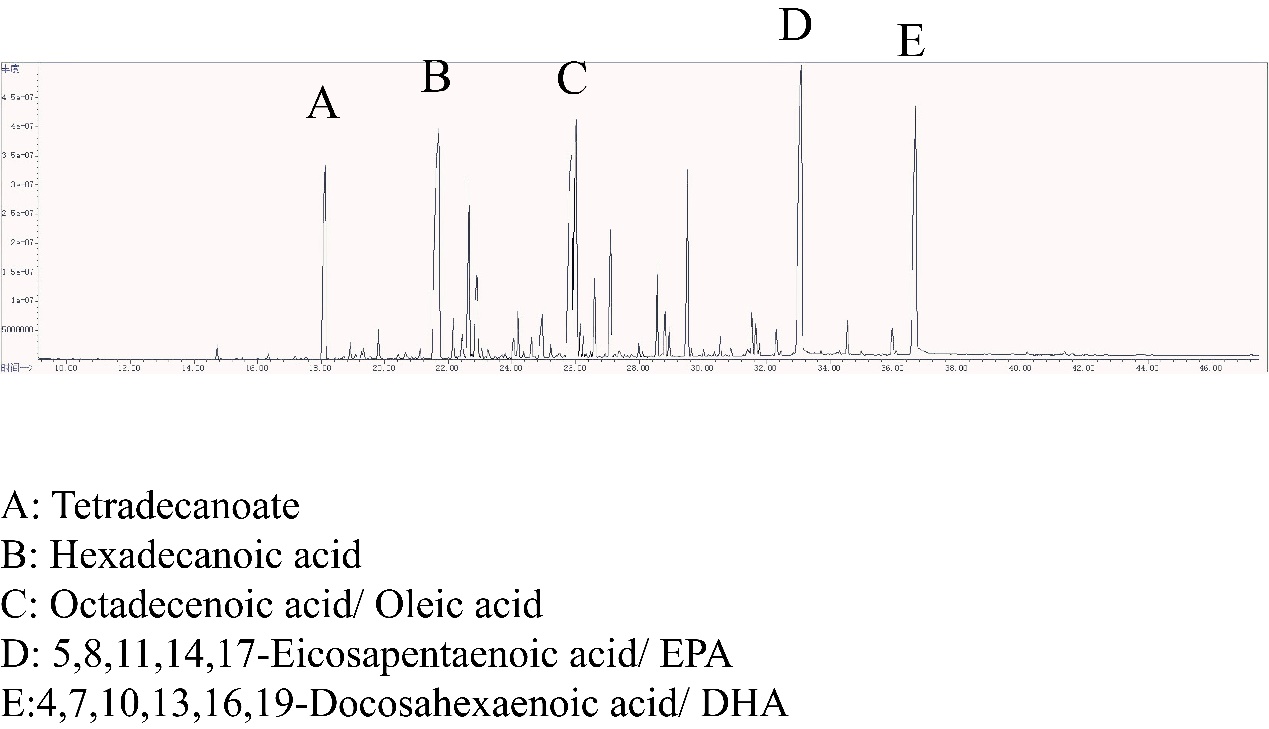
Figure S1 Characterization of SFE-extracted fatty acids by GC-MS

1. Spectrum of four tocopherol standard solutions

1. Spectrum of Krill oil

7.866

AU

0.00

0.02

0.04

0.06

0.08

0.10

0.12

0.14

0.16

0.18

0.20

0.22

0.24

·ÖÖÓ

0.00

1.00

2.00

3.00

4.00

5.00

6.00

7.00

8.00

9.00

10.00

11.00

12.00

13.00

14.00

15.00

16.00

17.00

18.00

1. α- Tocopherol curve（4.29~85.8 ug/mL）： Y=3.777×10^3^-3.954×10^2^ R^2^=0.9996

1. β- Tocopherol curve（1.70~34.1 ug/mL）： Y=4.479×10^3^-1.352×10^3^ R^2^=0.9991

1. γ- Tocopherol curve（2.14~42.8 ug/mL）： Y=4.351×10^3^-1.484×10^2^ R^2^=0.9998

1. δ- Tocopherol curve（4.29~85.8 ug/mL）： Y=3.816×10^3^-1.341×10^3^ R^2^=0.9998

Figure S2 Spectrum and stander curve of tocopherol

1. VA Spectrum of standard solution

1. Spectrum of Krill oil

1. VA standard curve（0.655~13.1 ug/mL）： Y=1.0194×10^5^+3.071×10^3^ R^2^=0.9999

Figure S3pectrum and stander curve of VA

Table S1 Primers used for qPCR analysis

| Gene | Primer sequence 5′–3′ |
| --- | --- |
| GAPDH-F | CCAGAGCTGAACGGGAAGCTCAC |
| GAPDH-R | CCATGTAGGCCATGAGGTCCACC |
| IL-1β-F | CTTCAGGCAGGCAGTATCAC |
| IL-1β-R | CAGCAGGTTATCATCATCATCC |
| IL-6-F | CTGCAAGAGACTTCCATCCAG |
| IL-6-R | AGTGGTATAGACAGGTCTGTTGG |
| TNF-α-F | CTGTGAAGGGAATGGGTGTT |
| TNF-α-R | CAGGGAAGAATCTGGAAAGGTC |

Table S2 Measurement results of injury score index, injury inhibition rate, and incidence of gastric injury in SD rat with ethanol stimulation after gavaging 100 mg/kg, 200 mg/kg, 500 mg/kg of oil for 30 d

| Group | Incidence of gastric injury (%) | Injury score index | Injury inhibition rate (%) |
| --- | --- | --- | --- |
| Control | 0 | 0 | 100 |
| Model | 65.25 ± 3.25^a^ | 46 ± 4^a^ | 0 ^a^ |
| KOL | 50.56 ± 4.68^b^ | 34 ± 3^b^ | 26.09 ± 2.56 ^b^ |
| KOM | 40.26 ± 3.58^b^ | 26 ± 2^b^ | 43.48 ± 5.69 ^b^ |
| ­KOH | 30.69 ± 2.98^b^ | 21 ± 2^b^ | 54.34 ± 4.58 ^b^ |

^1^Values (n = 6; mean ± SEM) different letters indicate statistically significant differences from the same column model group (*p< 0.05*). KOL: krill oil of 100 mg/kg, KOM: krill oil of 200 mg/kg, KOH: krill oil of 500 mg/kg
